# Calibration of a Heterogeneous Brain Model Using a Subject-Specific Inverse Finite Element Approach

**DOI:** 10.3389/fbioe.2021.664268

**Published:** 2021-05-04

**Authors:** J. Sebastian Giudice, Ahmed Alshareef, Taotao Wu, Andrew K. Knutsen, Lucy V. Hiscox, Curtis L. Johnson, Matthew B. Panzer

**Affiliations:** ^1^Center for Applied Biomechanics, University of Virginia, Charlottesville, VA, United States; ^2^Department of Electrical and Computer Engineering, Johns Hopkins University, Baltimore, MD, United States; ^3^Center for Neuroscience and Regenerative Medicine, The Henry M. Jackson Foundation for the Advancement of Military Medicine, Bethesda, MD, United States; ^4^Department of Biomedical Engineering, University of Delaware, Newark, DE, United States

**Keywords:** traumatic brain injury, material properties, magneticresonance elastography, image registration, morphing

## Abstract

Central to the investigation of the biomechanics of traumatic brain injury (TBI) and the assessment of injury risk from head impact are finite element (FE) models of the human brain. However, many existing FE human brain models have been developed with simplified representations of the parenchyma, which may limit their applicability as an injury prediction tool. Recent advances in neuroimaging techniques and brain biomechanics provide new and necessary experimental data that can improve the biofidelity of FE brain models. In this study, the CAB-20MSym template model was developed, calibrated, and extensively verified. To implement material heterogeneity, a magnetic resonance elastography (MRE) template image was leveraged to define the relative stiffness gradient of the brain model. A multi-stage inverse FE (iFE) approach was used to calibrate the material parameters that defined the underlying non-linear deviatoric response by minimizing the error between model-predicted brain displacements and experimental displacement data. This process involved calibrating the infinitesimal shear modulus of the material using low-severity, low-deformation impact cases and the material non-linearity using high-severity, high-deformation cases from a dataset of *in situ* brain displacements obtained from cadaveric specimens. To minimize the geometric discrepancy between the FE models used in the iFE calibration and the cadaveric specimens from which the experimental data were obtained, subject-specific models of these cadaveric brain specimens were developed and used in the calibration process. Finally, the calibrated material parameters were extensively verified using independent brain displacement data from 33 rotational head impacts, spanning multiple loading directions (sagittal, coronal, axial), magnitudes (20–40 rad/s), durations (30–60 ms), and severity. Overall, the heterogeneous CAB-20MSym template model demonstrated good biofidelity with a mean overall CORA score of 0.63 ± 0.06 when compared to *in situ* brain displacement data. Strains predicted by the calibrated model under non-injurious rotational impacts in human volunteers (*N = 6*) also demonstrated similar biofidelity compared to *in vivo* measurements obtained from tagged magnetic resonance imaging studies. In addition to serving as an anatomically accurate model for further investigations of TBI biomechanics, the MRE-based framework for implementing material heterogeneity could serve as a foundation for incorporating subject-specific material properties in future models.

## Introduction

Traumatic brain injury (TBI) is a significant source of injury, disability, and death. Recent epidemiological studies have estimated that TBI’s account for approximately one-third of all injury-related deaths in the United States (Faul and Coronado, 2015). In 2010, the Centers for Disease Control (CDC) estimated that TBIs resulted in 2.5 million emergency department (ED) visits (87%), hospitalizations (11%), and deaths (2%) (Centers for Disease Control and Prevention, 2015). Finite element (FE) models of the brain have rapidly become indispensable tools for investigating TBI mechanisms, assessing new protective technology, and developing injury risk criteria (Kleiven and von Holst, 2002; Giordano and Kleiven, 2014; Gabler et al., 2018). FE models of the brain are typically used to investigate the dynamic 3D deformation of the human brain under simulated head impacts relevant to sports, automotive crashes, and falls. While FE models have been instrumental to furthering our understanding of TBI biomechanics, many FE brain models have been developed to represent the brain as a simplified physical system, both in their representation of the anatomy and material properties, thus limiting their accuracy and utility in predicting deformations experienced due to head impacts.

Perhaps the most significant differences across brain models in the field relate to the constitutive laws and material parameters chosen to represent the material behavior of the simulated brain tissue (Jin et al., 2013; Dixit and Liu, 2017; Fahlstedt et al., 2021). At the simplest level, the brain is modeled as a single isotropic and homogeneous material (Kleiven and von Holst, 2002; Takhounts et al., 2008; Ji et al., 2015). However, unique material properties can be assigned to different parts of the brain, which typically represent tissue types with different cellular composition or segmented anatomical labels, and many FE models include differences in material properties between white and gray matter (Horga and Gilchrist, 2003; Kimpara et al., 2006; McAllister et al., 2012; Panzer et al., 2012; Mao et al., 2013; Miller et al., 2016, 2017). At the most complex level, the brain has been modeled as an anisotropic, heterogeneous structure by explicitly modeling axonal fiber tracts by embedding 1D elements in the brain mesh (Garimella et al., 2019; Hajiaghamemar et al., 2019; Wu et al., 2019). While the embedded axon approach provides a more biofidelic structural representation of the axonal tissue, which is known to exhibit anisotropy and regional variations in tissue material properties (Jin et al., 2013; Budday et al., 2015, 2017a, 2019; Weickenmeier et al., 2016), the embedding of 1D axonal tract elements can significantly increase computational cost. A potential alternative for modeling brain heterogeneity is to model the brain with material stiffness varying throughout the brain regardless of the tissue classification of each element, which can be obtained using magnetic resonance elastography (MRE).

Recently, MRE has been utilized to non-invasively measure *in vivo* material properties of the human brain in healthy volunteers (Weaver et al., 2012; Johnson et al., 2013a, b; Hiscox et al., 2016). In MRE, an external transducer (commonly a head pillow) mechanically vibrates the head (10–100 Hz) to induce micron-level displacements in the brain that can be measured and used to estimate elastic and viscous material properties throughout the brain with high spatial resolution (Hiscox et al., 2016). To date, MRE has been used to investigate global brain material properties with voxel-level resolution, regional variations in tissue stiffness, and material properties associated with brain pathology (Hiscox et al., 2016; Johnson and Telzer, 2018; Murphy et al., 2019). While stiffness measurements vary, most studies agree that the measured brain stiffness is dependent on the actuation frequency (due to viscoelasticity), and that white matter regions are stiffer than gray matter regions (Hiscox et al., 2016). However, since the measured properties are dependent on the actuation frequency and obtained from micron-level displacements of the brain, additional work is needed to apply the MRE-derived stiffness maps to a FE brain model.

The goal of this study is to calibrate and verify a heterogenous FE brain model by leveraging experimental datasets reporting (1) MRE-derived material properties (Hiscox et al., 2020), (2) high rate, *in situ*, brain displacements measured from human cadaveric specimens using sonomicrometry (Alshareef et al., 2020a), and (3) low rate, *in vivo*, brain strain measured from human volunteers using tagged magnetic resonance imaging (tMRI; Knutsen et al., 2020). Material parameters for the model were developed in three phases. First, stiffness data from an MRE template image (average of 134 subjects; Hiscox et al., 2020) was used to define the relative stiffness gradient throughout the brain model. Second, the linear stiffness parameter was calibrated using low-displacement cases from the Alshareef et al. (2020a) dataset and verified using brain strain data from the *in vivo* tMRI dataset. Finally, the non-linear stiffness parameter was calibrated using high-deformation cases from the Alshareef et al. (2020a) dataset. To verify that the calibrated material parameters were physically reasonable, a comprehensive verification was performed using the remaining rotational cases from these datasets that were not used for calibration. The response of the calibrated material used in the model was also compared to experimental *in vitro* material test data available in the literature (Jin et al., 2013) to ensure that the median stiffness response was within the range of experimental data. In addition to serving as a model for investigating TBI biomechanics, the technique for implementing MRE-derived heterogeneous material properties can be adapted to implement subject-specific material properties in future subject-specific brain models.

## Materials and Methods

### Development of CAB-20MSym Template Model

The mesh of the CAB-20MSym template model was chosen to represent the anatomy of the CAB-20MSym template image developed by Giudice et al. (2020). This template was constructed from T1-weighted MRI scans obtained from 20 young, healthy adult males (22 ± 3 years). Details regarding these images are provided elsewhere (Giudice et al., 2020; Reynier et al., 2020).

The CAB-20MSym template image was segmented to identify the brain parenchyma, peripheral cerebrospinal fluid (CSF), internal CSF, and ventricles. To generate the template model mesh, each 1 mm isotropic voxel in the segmentation image was directly converted into a cubic hexahedral (i.e., voxel) element and assigned to a part based on its segmentation label. This approach was selected as voxel meshes have the accuracy and stability benefits of hexahedral elements and can capture complex anatomical features at the native spatial resolution (in this case, 1 mm) of the MRI images used to construct them (Miller et al., 2016; Ghajari et al., 2017). To include the sagittal sinus, falx cerebri, and tentorium cerebelli, the voxels surrounding these regions were manually delineated and two-dimensional shell elements were generated at the corresponding mid-surfaces. Finally, a layer of rigid shell elements surrounding the outermost surface of the peripheral CSF part was generated to represent the dura, which was assumed to be rigidly connected to the inner surface of the skull (Miller et al., 2016). The selection of the numerical implementation approach was informed by an analytical review of the numerical methods utilized by the TBI modeling community performed by Giudice et al. (2019b) and is summarized in the Supplementary Material. All interfaces were continuous and connected through shared nodes.

The material properties for the CSF (peripheral and internal), ventricles, skull, sagittal sinus, falx, and tentorium were adapted from previous brain models (Takhounts et al., 2008; Mao et al., 2013; Miller et al., 2016). To account for the nominal stiffness provided by the trabeculae and bridging vessels located within the subarachnoid space, the peripheral CSF was modeled using a linear viscoelastic material with very low stiffness (*G*_*0*_ = 0.5 kPa; *G*_*∞*_ = 0.1 kPa). As these properties do not exist for CSF, an elastic fluid (bulk modulus, *K* = 2.1 GPa) was assigned to the ventricle and internal CSF parts. The sagittal sinus, falx, and tentorium were modeled as elastic materials (Young’s modulus, *E* = 31.5 MPa; Poisson’s ratio, ν = 0.45). Finally, the skull was modeled as rigid to allow the implementation of 6 degree-of-freedom head kinematic boundary conditions for all analyses (Gabler et al., 2016). Further details regarding these material properties are available in the Supplementary Material. The material implementation of the brain parenchyma is described in Sections “Implementation of Brain Heterogeneity” and “Constitutive Modeling of Brain Parenchyma.”

### Implementation of Brain Heterogeneity

The implementation of brain material heterogeneity in the CAB-20MSym template model was derived from the MRE134 template image (Hiscox et al., 2020). This template was constructed using MRE data from 134 healthy, young adults (18–35 years, 78F/56M) using common MRE acquisition and data processing protocols (Hiscox et al., 2020). To adapt the MRE134 template, originally defined in MNI152 space with 2 mm isotropic voxels, it was first non-linearly registered to the CAB-20MSym template space using ANTs non-linear registration (Avants et al., 2008). To eliminate stiffness measurements potentially influenced by numerical artifacts or edge effects, stiffness values beyond the 98th percentile were excluded. In the CAB-20MSym template model, CSF spaces were modeled using CSF-specific constitutive models, however, in the MRE134 template image, CSF spaces were not differentiated from the brain tissue. Therefore, stiffness values below the 15th percentile, which corresponded to the approximate stiffness in the MRE134 template in CSF areas (approximately 0–1.5 kPa; Hiscox et al., 2020), were excluded. From this truncated distribution, the stiffness value of each voxel was normalized by the median stiffness and binned into 10 groups. Voxels that had original stiffnesses below the 15th and above the 98th percentile were assigned to the lowest and largest normalized stiffness bins, respectively. This process yielded a normalized stiffness label image where voxels were categorized by their relative stiffness, and not according to an anatomical segmentation label. In doing so, this approach accounts for stiffness variations present within tissue groups, such as white and gray matter. Normal variations in the material properties of various tissue groups are reported in the literature (Hiscox et al., 2020).

### Constitutive Modeling of Brain Parenchyma

The 10 parts comprising the brain parenchyma, using the binned groups from the previous section, were modeled using a quasi-linear viscoelastic (QLV) model (Fung, 1993). In Fung’s QLV theory, it is assumed that the response of a material can be separated into a normalized function of time only, *g*(*t*), and an elastic function of strain only, *T*^*e*^(ε) (Fung, 1993). For a QLV model, the stress relaxation function, *R*(ε,*t*), is:

$$R\,\left(\varepsilon ,t\right)=g\,\left(t\right)\cdot T^{e}\,\left(\varepsilon \right)$$

In this study, the instantaneous elastic response function, *T*^*e*^(ε), was derived from an Ogden strain energy density (Ogden and Hill, 1972).

$$W\,\left(\lambda_{1},\lambda_{2},\lambda_{3}\right)=\frac{\mu }{\alpha }\,\left(\lambda_{1}^{\alpha }+\lambda_{2}^{\alpha }+\lambda_{3}^{\alpha }-3\right)$$

Where λ_*j*_ are the three principal stretches, μ is the shear modulus, and α is a unitless non-linearity coefficient. The infinitesimal shear modulus (i.e., initial slope of the non-linear shear stress-strain curve), μ_*0*_, can be obtained as a function of the material parameters.

$$\mu_{0}=\frac{1}{2}\,\mu \,\alpha$$

Finally, four Prony terms (*N* = 4) were included in the reduced relaxation function.

$$g\,\left(t\right)=g_{\infty }+\sum_{i=1}^{N}g_{i}\,e^{-\beta_{i}\,t}$$

$$\beta_{i}=\frac{1}{\tau_{i}}$$

$$g_{\infty }+\sum_{i=1}^{N}g_{i}=1$$

Where, *g*_*∞*_ and *g*_*i*_ are normalized coefficients associated with the long-term response and each time constant, τ_*i*_. The density, ρ, and Poisson’s ratio, ν, of the brain parenchyma material were 1.123 × 10^–6^ kg/mm^3^ (Miller et al., 2016) and 0.499999, respectively.

The reduced relaxation function was fit to experimental tan(δ) data obtained from studies that characterized the viscoelastic properties of brain tissue over wide ranges of input frequencies (Fallenstein et al., 1969; Shuck and Advani, 1972; Arbogast et al., 1997; Bilston et al., 1997, 2001; Arbogast and Margulies, 1998; Brands, 2000; Darvish and Crandall, 2001; Lippert et al., 2004; Nicolle et al., 2004; Hrapko et al., 2006; Shen et al., 2006; Garo et al., 2007). The tan(δ) response represents material damping as a function of frequency, and is independent of the material stiffness, making it suitable for calibrating the reduced relaxation function. Optimization of *g*_*i*_ and τ_*i*_ was performed using a least squares optimization. The Ogden parameters defining the median deviatoric response of the brain, to which the brain parenchyma heterogeneity was applied relative to, was calibrated using an iFE calibration scheme and is described in the following sections.

### Calibration Objective and Approach

To reduce the number of optimized parameters and simplify the calibration process, it was assumed that the damping of the brain and hyperelastic non-linearity were homogeneous throughout the brain. As such, the reduced relaxation function parameters were assigned to all brain parenchyma parts. Therefore, the objective of this inverse FE approach was to calibrate two parameters:

1. The median stiffness of the brain, μ_*med*_, that defined the material heterogeneity.
2. The non-linearity coefficient, α, that defined the hyperelastic non-linearity.

Material parameters were calibrated using an iFE approach in which parameters were optimized to minimize the error between model and experimental results. In this case, a subset of the impact cases in the *in situ* brain displacement dataset (Alshareef et al., 2018, 2020a, b) were simulated using subject-specific models and the error between nodal displacements and the corresponding experimental displacements were minimized. In this dataset, *in situ* brain deformation was measured at discrete locations using sonomicrometry sensors (i.e., “receivers”), distributed throughout the brain parenchyma of cadaveric head-neck specimens. Three subject-specific models were generated to represent the anatomies of subjects SONO-896, SONO-900, and SONO-904 (Table 1; Alshareef et al., 2020a). Further details regarding this experimental dataset and the corresponding model setup are provided in the Supplementary Materials.

**TABLE 1:** Specimen information for subjects used to assess deformation response (Alshareef et al., 2020a).

Specimen	SONO-896	SONO-900	SONO-904
Axial MRI	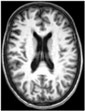	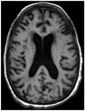	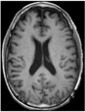
Sex	Female	Female	Male
Age (yrs)	57	66	67
Height (cm)	163	165	177
Body Mass (kg)	31.1	56.2	54.9
ICV (cm^3^)	1300	1406	1545

To minimize the likelihood of obtaining a non-unique solution, μ_*med*_ and α were optimized independently by leveraging specific cases in the *in situ* brain displacement database. To optimize μ_*med*_ a low severity case (Axial 20 rad/s, 60 ms) was used to optimize the median infinitesimal quasi-static shear stiffness (a function of μ_*med*_). This case was selected as preliminary simulations indicated that the deformations induced in this case were not sensitive to the material non-linearity coefficient. A higher severity case (Coronal 40 rad/s, 30 ms) was used to optimize α while maintaining the optimized infinitesimal quasi-static shear stiffness. This coronal rotation case was selected as preliminary simulations indicated that the deformations predicted in this case were sensitive to the material non-linearity coefficient and avoided calibrating the material parameters under a single loading direction. Finally, the calibrated heterogeneous material parameters were verified using the remaining cases in the *in situ* brain deformation database, as well as low severity *in vivo* brain strain data obtained using the *in vivo* tMRI database (Knutsen et al., 2020). This final verification included 39 simulations; 11 simulations for each SONO subject-specific model (not including case used for calibration) and 6 simulations for the tMRI cases. Details regarding these experimental datasets and the respective model setups are provided in the Supplementary Materials. As a final check, the material response was assessed in tension, compression, and simple shear loading over strain rates of 0.5, 5, and 30 1/s and compared to experimental data (Jin et al., 2013).

The material calibration process is summarized below:

1. Optimize μ_*med*_ under low severity rotational impacts.
2. Intermediate verification to assess calibrated μ_*med*_ under independent low severity cases.
3. Optimize α under higher severity rotational impacts.
4. Comprehensive verification of calibrated material parameters

All models used in this study were subject-specific, constructed to represent the anatomies of the subjects from which the experimental data were collected. As such, the deformation response of each subject-specific model was compared to the experimental data obtained from that subject only. This was crucial to eliminate geometric effects from the calibration and verification processes, ensuring that discrepancies between simulation and experimental results, for each subject, were due to variations in material parameters only. All subject-specific models were generated using registration-based morphing (RBM), which is a non-linear morphing technique that was developed specifically for generating subject-specific models of the brain by leveraging image registration transformations (Giudice et al., 2020). Metrics of morphing accuracy and element quality for all subject-specific models generated in this study are provided in the Supplementary Materials.

### Objective Rating and Optimization Strategy

CORrelation and Analysis (CORA) scores were used to quantify the error between the nodal displacements in the model and the receiver displacements in the experimental dataset. Since experimental displacements were 3D (i.e., x, y, and z displacement time-histories for each receiver), a composite score (cCORA) was calculated to obtain a single objective rating for each receiver. cCORA was computed as the weighted average of the CORA scores in each orthogonal direction, weighted by the relative magnitude of the experimental signal in each direction (Giudice et al., 2019a). Finally, the overall score for each rotational case was computed as the weighted average of all receiver cCORA scores, weighted by the experimental maximum resultant displacement for each receiver (wcCORA). In this study, the default CORA parameters were used (Gehre et al., 2009).

$$w\,c\,C\,O\,R\,A=\sum_{i}^{N}\alpha_{i}\times c\,C\,O\,R\,A_{i}$$

$$\alpha_{i}=\frac{\beta_{\text{i}}}{\sum_{i}^{N}\beta_{\text{i}}}$$

Where, β_i_ is the experimental maximum resultant displacement for the *i*^*th*^ receiver and *N* is the number of receivers for each rotation case. For the calibration of the Ogden material parameters, wcCORA was used to quantify the error between the nodal displacements and the experimental receiver displacements for each rotation simulation. To obtain a set of parameters that best represented the overall response of the three subjects used for calibration, a joint optimization was performed where the goal was to maximize the mean wcCORA score for subjects SONO-896, SONO-900, and SONO-904.

$$f\,\left(x\right)=\frac{w\,c\,C\,O\,R\,A_{896}+w\,c\,C\,O\,R\,A_{900}+w\,c\,C\,O\,R\,A_{904}}{3}$$

A golden ratio search algorithm was used to identify the maximum mean overall wcCORA as a function of the material parameter being optimized. In the first iteration, a series of simulations were run to identify the bounds for the golden search algorithm. For example, when calibrating α, three simulations with α = 2, 6, and 10 were run to determine the bounds for calibration. The parameters investigated in each subsequent iteration, *i* (*x*_*i,1*_ and *x*_*i,2*_) were determined by the golden ratio search algorithm.

$$x_{i,1}=a_{i}+\left(1-\varphi \right)\,\left(b_{i}-a\right)$$

$$x_{i,2}=a_{i}+\varphi \,\left(b_{i}-a\right)$$

$$\varphi =\frac{\sqrt{5}-1}{2}$$

Where *a*_*i*_ and *b*_*i*_ are the lower and upper bounds for iteration, *i*. The bounds were updated based on the mean wcCORA score for each parameter investigated [*f*(*x*_1_) and *f*(*x*_2_)] in the previous iteration (*i* – 1).

$$\mathrm{If}\,f\,\left(x_{1}\right)>f\,\left(x_{2}\right)\,\mathrm{then}\,a_{i}=a_{\left(i-1\right)}\,\mathrm{and}\,b_{i}=x_{\left(i-1\right),2}$$

$$\mathrm{If}\,f\,\left(x_{1}\right)<f\,\left(x_{2}\right)\,\mathrm{then}\,a_{i}=x_{\left(i-1\right),1}\,\mathrm{and}\,b_{i}=b_{\left(i-1\right)}$$

This process was repeated until the termination criteria was satisfied. The parameter that had the greatest mean wcCORA was selected as the calibrated value. The first iteration and termination criteria for the calibration of μ_*med*_ and α are shown in Table 2. These values were selected based on parameters reported in the literature for human brain tissue (Miller and Chinzei, 2002; Nicolle et al., 2004; Franceschini et al., 2006; Kleiven, 2007; Kaster et al., 2011; Moran et al., 2014; Budday et al., 2017a).

**TABLE 2:** First iteration parameters and termination criteria for calibrated material parameters.

Parameter	1st Iteration	Termination Criteria
μ_*med*_	μ_*med*_ = 0.25, 0.7, 1.15, 1.6, 2.05, 2.6 kPa	*b*_(*i* + 1)_−*a*_(*i* + 1)_ < 0.1*kPa*
α	α = 2, 6, 10	*b*_(*i* + 1)_−*a*_(*i* + 1)_ < 0.2

### Calibration and Verification of Median Shear Modulus

In the first step of the material calibration process, μ_*med*_ was optimized by simulating the Axial 20 rad/s, 60 ms case (Z: 20–60). All simulations were run for 200 ms. Pilot simulations indicated that the predicted deformations in this loading case were not sensitive to material non-linearity that is governed by α. Therefore, in this optimization the shear response of the material was constrained to the linear response of a Neo-Hookean solid (α = 2), where the shear modulus defined in the Ogden model is identical to the infinitesimal shear modulus of the material (μ_*0*_).

Since the model incorporates material heterogeneity relative to the median stiffness of the brain, the stiffness of each of the 10 parts was defined relative to the median stiffness defined for any given optimization iteration.

$$\mu_{i}=\gamma_{i}\,\mu_{m\,e\,d}=\gamma_{i}\,\mu_{0,m\,e\,d}$$

Where γ_*i*_ is the relative stiffness of the *i*th part and varies between 0.53 and 1.53. All stiffness values reported in this study refer to quasi-static parameters (i.e., μ_*med*_ and α represent the quasi-static response of the material).

To verify the calibrated median shear stiffness (μ_0,*med*_ = μ_*med*_) the remaining 20 rad/s, 60 ms cases in the coronal (X: 20–60) and sagittal (Y: 20–60) directions were simulated and compared to each specimen’s experimental data using wcCORA. Verification of the strain response was performed by simulating *in vivo* brain strain experiments (Knutsen et al., 2020). In this dataset, three subjects (tM-3978, tM-4838, and tM-6176) were subjected to sagittal rotations (ω_*max*_ = 1.4–1.6 rad/s) and three subjects (tM-3978, tM-7126, and tM-9475) were subjected to axial rotations (ω_*max*_ = 4–5.4 rad/s) of the head. This verification step was performed to ensure that the calibrated μ_*0,med*_ was physically meaningful and resulted in biofidelic deformation predictions under independent low-deformation test cases. This also provided reassurance that the final set of calibrated material parameters were unique, given the model’s sensitivity to both μ and α.

To compare the predicted strain response of each subject-specific model, the maximum principal strain (MPS) of each element was computed and mapped to the corresponding voxel in the subject image. As a global metric of strain, the 95th percentile MPS value (MPS-95) predicted by the model was compared to the equivalent experimental measures. In addition, the volume fraction of elements exceeding 2% strain was compared between the model and experimental data. These volume fractions were computed globally, as well as regionally for the cerebral gray and white matter and the cerebellum. These regions were identified using a segmentation image provided in the tMRI database. MPS-95 was also computed for the elements/voxels located in these regions.

### Calibration and Verification of Non-linear Coefficient

In the previous steps, the median shear stiffness, which was equivalent to the median infinitesimal shear modulus (μ_0,*med*_ = μ_*med*_) since the shear response was assumed to be linear (α = 2), was determined and verified. In this step, the non-linear coefficient (α) was calibrated by simulating the Coronal 40 rad/s, 30 ms rotation case. All simulations were run for 200 ms. To preserve the previously calibrated μ_*0,med*_, μ_*med*_ was also adjusted such that μ_*0,med*_ was equivalent to the value determined in the first step of the optimization procedure. Therefore, for the *i*^*th*^ part in the heterogeneous CAB-20MSym template model the shear modulus in the Ogden constitutive model, μ_*i*_, was defined as a function of μ_*med*_, α, and γ_*i*_.

$$\mu_{i}=\frac{2\,\gamma_{i}\,\mu_{0,m\,e\,d}}{\alpha }$$

To verify the calibrated heterogeneous material, the remaining 11 rotation cases for each subject were simulated (36 total simulations including case used for calibration), and the nodal displacements of the calibrated subject-specific models were compared to the corresponding experimental brain displacement data using wcCORA. To assess the calibrated model performance relative to other state-of-the-art and widely used FE brain models, wcCORA values obtained in this study were compared to those reported for the Global Human Body Models Consortium (GHBMC) brain model (Mao et al., 2013) and the UVA embedded axon model (UVA-EAM) (Wu et al., 2019, 2020, 2021). These models were morphed to the anatomy of the three subjects using surface-based morphing (Wu et al., 2019) and simulated under identical boundary conditions, resulting in 36 simulations per model.

The set of tMRI simulations used to verify the calibrated median infinitesimal shear stiffness were also simulated using the final calibrated heterogeneous model to ensure that the incorporation of material non-linearity did not influence the model predictions. Since the brain strains in these simulations were low (less than 6% MPS), material non-linearity was not expected to influence the model results.

Finally, the calibrated Ogden material response was compared to experimental material test data. To verify the optimized parameters, the complex modulus of the derived model was compared to the complex moduli reported in the rheological characterization dataset. Furthermore, a series of single element (1 mm × 1 mm × 1 mm) simulations were run with the fit material parameters to verify the response of the constitutive model as well as the Ogden QLV implementation in LS-Dyna. These single element simulations were run in tension, compression, and simple shear at loading rates of 0.5, 5, and 30 1/s to 50% engineering strain and the results were compared to average response corridors constructed from the material characterization data in the literature (Jin et al., 2013).

## Results

### Development of CAB-20MSym Template Model

**FIGURE 1:**
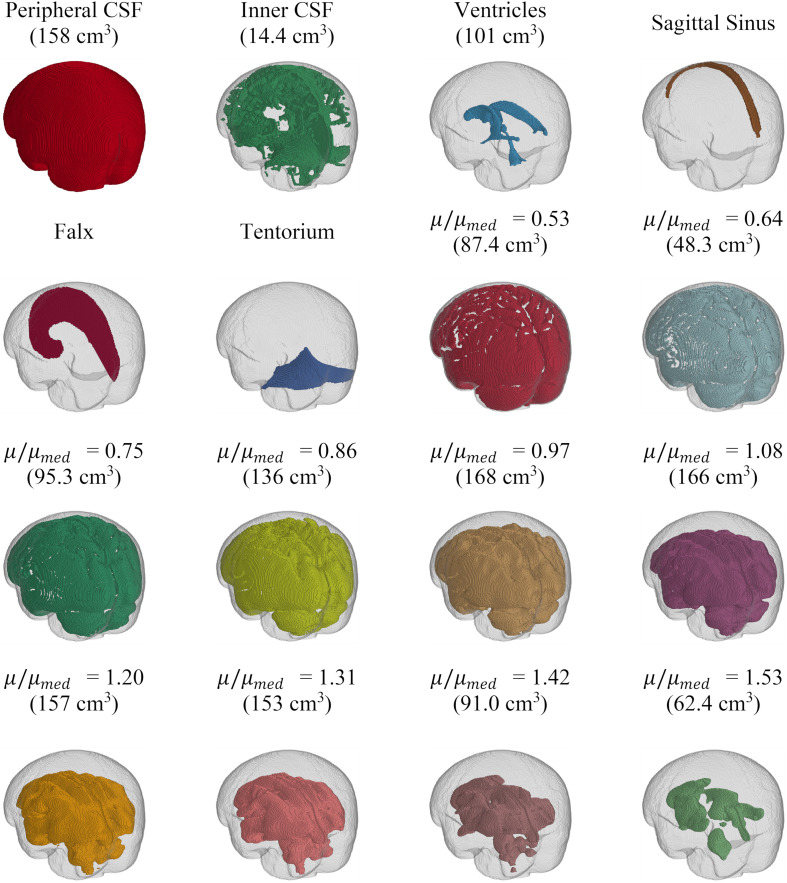
Depiction of the parts of the CAB-20MSym mesh. Brain parenchmya parts are subdivided based on their stiffness relative to the median stiffness (μ/μ_*med*_) of the MRE134 template. The volume of each solid part is indicated in parentheses. The skull is shown in gray.

The CAB-20MSym template model had an intracranial volume of 1439 cm^3^ and approximately 1.6 million elements, 1.5 million nodes, and 16 parts (Figure 1). Note that Figure 1 depicts the external surfaces of each of these parts and is not representative of the entire volume of each part. All interfaces (e.g., falx-brain) were continuous and defined using shared nodes.

### Implementation of Brain Heterogeneity

**FIGURE 2:**
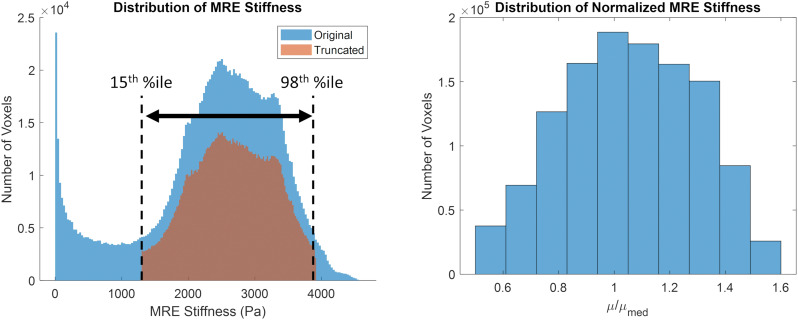
**(Left)** original (blue) and truncated (orange) MRE134 stiffness distributions. Stiffness values in the original distribution were truncated between the 15th and 98th percentile stiffness values. **(Right)** grouped normalized stiffness values (*N* = 10 bins).

**FIGURE 3:**
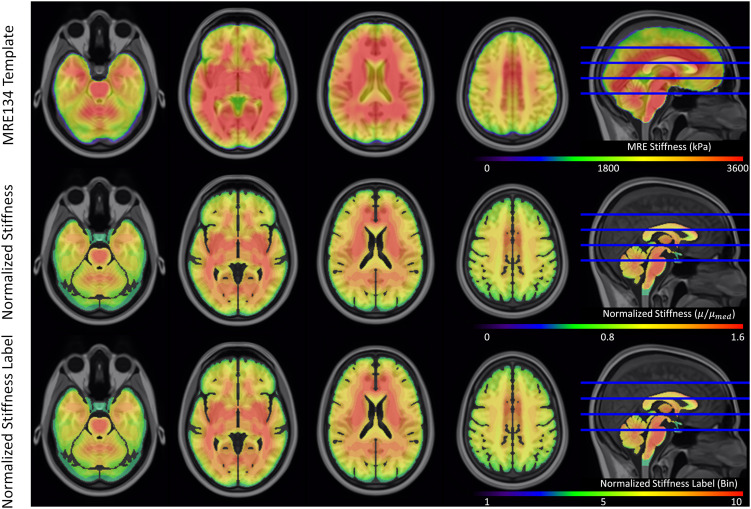
Magnetic resonance elastography template (MRE134), normalized stiffness image, and normalized stiffness label image (used to construct CAB-20MSym template model). Each of the 10 bins in the normalized stiffness label corresponded to an individual part in the template model.

The original and truncated stiffness distributions from the MRE134 template, mapped to CAB-20MSym space, are shown in Figure 2. In the truncated distribution, the median stiffness was 2.53 kPa and the mean ± standard deviation was 2.37 ± 0.99 kPa. To convert the MRE134 template image into the CAB-20MSym template model, the truncated distribution was grouped into 10 bins, which were used to classify the normalized stiffness of each voxel in the CAB-20MSym model. The 10 relative stiffness groups were 0.53, 0.64, 0.75, 0.86, 0.97, 1.08, 1.20, 1.31, 1.42, and 1.53, which corresponded to the bin centers (Figure 2). The MRE134 template, normalized stiffness image, and normalized stiffness label image are shown in Figure 3.

### Constitutive Modeling of the Brain Parenchyma

**FIGURE 4:**
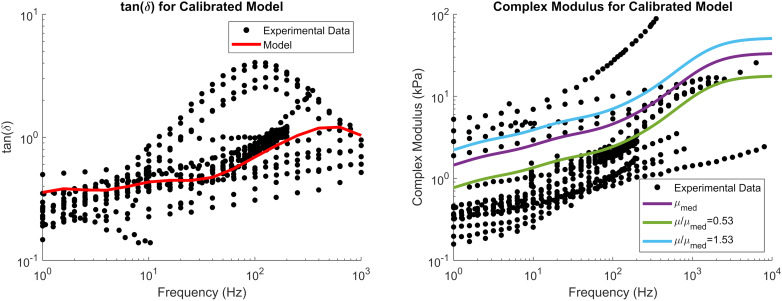
tan⁡(δ) **(left)** and complex modulus **(right)** of the calibrated material parameters, demonstrating the median complex modulus of the brain (purple) as well as the complex modulus for the softest (green) and stiffest (blue) relative stiffness parts. Note that tan⁡(δ) is a function of damping only and not tissue stiffness. Experimental data were obtained from the literature (see Section “Constitutive Modeling of Brain Parenchyma” for list of references).

The reduced relaxation function parameters were obtained by fitting *g*_*i*_ and β_*i*_ (*i* = 4) to match experimental tan⁡(δ) data (Figure 4). The fit reduced relaxation parameters are shown in Table 3. These viscoelastic parameters were applied homogeneously throughout the entire brain model.

**TABLE 3:** Prony series parameters for the brain parenchyma.

	Material Parameters		
	*g*_1_ = 0.8619		β_1_ = 10*ms*
	*g*_2_ = 0.0383		β_2_ = 1*ms*
	*g*_3_ = 0.0412		β_3_ = 0.1*ms*
	*g*_4_ = 0.0249		β_4_ = 0.01*ms*
		*g*_∞_ = 0.0337	

### Calibration and Verification of Median Shear Modulus

**FIGURE 5:**
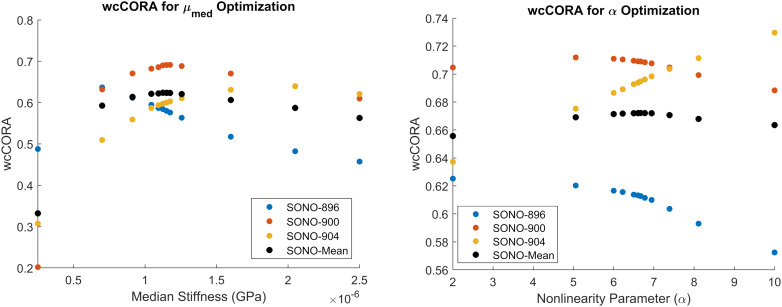
Calibration results for μ_*med*_ **(left)** and α **(right)**. Parameters were calibrated to maximize the mean wcCORA score (black) across subjects SONO-896 (blue), SONO-900 (red), and SONO-904 (yellow). wcCORA for each subject was obtained by comparing nodal displacements from corresponding subject-specific models and experimental data.

The median shear stiffness of the material was calibrated using the axial 20 rad/s, 60 ms rotation case in the *in situ* brain displacement database. The calibrated value of μ_*med*_ was 1.125 kPa. Since this initial constitutive model constrained the non-linear coefficient to 2 (representing a Neo-Hookean solid), μ_*med*_ was equivalent to the infinitesimal quasi-static shear modulus, μ_*0,med*_. Six iterations (12 simulations) were required to satisfy the termination criteria specified in Table 2, resulting in an optimal mean wcCORA of 0.62 (Figure 5). The calibrated value was within the range of other Ogden rubber infinitesimal quasi-static shear moduli reported in the literature for the brain (Miller and Chinzei, 2002; Nicolle et al., 2004; Franceschini et al., 2006; Kleiven, 2007; Kaster et al., 2011; Moran et al., 2014; Budday et al., 2017a), which varied from 0.27 to 1.49 (mean ± standard deviation = 0.92 ± 0.38).

Using the jointly calibrated μ_*med*_ = μ_0,*med*_ value, the Coronal and Sagittal 20 rad/s, 60 ms cases for the same specimens were simulated to verify the subject-specific model responses. The wcCORA scores for the coronal, sagittal, and axial simulations were between 0.66–0.72, 0.50–0.67, and 0.58–0.69, respectively (Table 4). In general, wcCORA scores were greatest in the coronal rotation, and across the three subjects, the SONO-900 model yielded the highest wcCORA scores using the jointly calibrated μ_*med*_ value.

**TABLE 4:** wcCORA scores for μ_*0,med*_ joint optimization and verification.

Subject	wcCORA		
	X: 20–60	Y: 20–60	Z: 20–60*
SONO-896	0.67	0.67	0.58
SONO-900	0.72	0.63	0.69
SONO-904	0.66	0.50	0.60
SONO-Mean	0.68 ± 0.03	0.60 ± 0.09	0.62 ± 0.06

*Axial 20 rad/s, 60 ms (Z: 20–60) case used for calibration.

**FIGURE 6:**
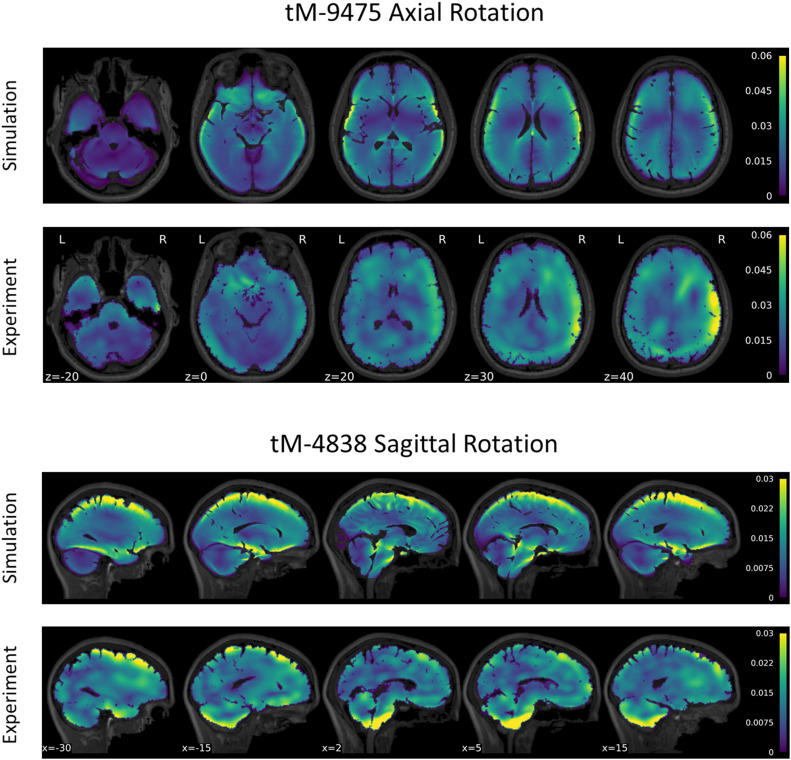
Comparison of maximum principal strain distribution for subject tM-9475 under axial rotation **(top)** and tM-4838 under sagittal rotation **(bottom)**.

A series of tMRI experiments were simulated using subject-specific models to verify the calibrated value of μ_*med*_ = μ_0,*med*_. The strain response of subject tM-9475 during the axial rotation is shown in Figure 6. Qualitatively, the tM-9475 subject-specific model demonstrated a similar deformation pattern compared to the experimental data. In general, MPS was largest in the cortex and smallest in the cerebellum (Figure 6). Additionally, in both simulation and experiment, larger strains were observed at the apexes of the ventricles (i.e., frontal and occipital horns) which propagated through the cerebrum. This can be seen in slices *z* = 20–40 in Figure 6. However, in the experimental data cortical strain was consistently asymmetric, with the largest strains observed in the right hemisphere of the brain, whereas in the simulation the MPS distribution was relatively symmetric. These trends were similar for all subjects tested in the axial rotation protocol.

These trends were salient in the MPS-95 and strain volume fraction metrics used to quantify brain deformation globally and regionally. For subject tM-9475, global and regional MPS-95 values were similar to the experimental data, with absolute differences between 0.0025 and 0.0084 strain. However, while regional MPS-95 values were similar between the model and experiment, larger differences in the volume fraction of voxels that exceeded 2% strain was observed, particularly in the cerebellum (simulation: 0.15; experiment: 0.33) and gray matter (simulation: 0.34; experiment: 0.61). Figures showing MPS-95 and volume fraction results for the axial and sagittal tMRI cases are included in the Supplementary Materials.

The strain response of subject tM-4838 during the sagittal rotation is shown in Figure 6. Qualitatively, the tM-4838 subject-specific model demonstrated a similar MPS pattern compared to the experimental data with the largest MPS values observed at the periphery of the brain, including the cortex, base of the cerebrum, and anterior surface of the brainstem, and lowest in the midbrain (Figure 6). However, strains in the cerebellum and brainstem were larger in the experimental data than predicted by the simulation. This is particularly evident in slices *x* = −15–5 in Figure 6. In general, global and regional MPS-95 metrics were similar between the subject-specific model and experimental data with absolute differences in MPS-95 between 0.0003 and 0.0067 for subject tM-4838. The volume fraction of voxels that exceeded 2% strain was also similar, except for the cerebellum, where the experimental volume fraction was 0.16 compared to only 0.06 in the simulation. These trends were similar for all subjects tested in the sagittal rotation protocol.

### Calibration and Verification of Non-linear Coefficient

The non-linearity parameter of the Ogden constitutive model, α, was calibrated using the Coronal 40 rad/s, 30 ms rotation case in the *in situ* brain deformation database. The calibrated value of α was 6.67, which yielded a mean wcCORA of 0.67 across subjects SONO-896, SONO-900, and SONO-904 (Figure 5). Eight iterations (12 simulations) were required to satisfy the termination criteria specified in Table 2. The final calibrated material parameters for each brain parenchyma part (grouped by stiffness relative to the median stiffness) are shown in Table 5. The viscoelastic parameters for the brain parts are shown in Table 3.

**TABLE 5:** Calibrated Ogden material parameters for each brain parenchyma part, grouped by stiffness relative to the median value.

Relative Stiffness	μ (kPa)	α
μ_*med*_^*a*^	0.337	6.67
μ/μ_*med*_ = 0.53	0.179	
μ/μ_*med*_ = 0.64	0.216	
μ/μ_*med*_ = 0.75	0.254	
μ/μ_*med*_ = 0.86	0.291	
μ/μ_*med*_ = 0.97	0.328	
μ/μ_*med*_ = 1.08	0.366	
μ/μ_*med*_ = 1.20	0.403	
μ/μ_*med*_ = 1.31	0.441	
μ/μ_*med*_ = 1.42	0.478	
μ/μ_*med*_ = 1.53	0.515	

*^*a*^Not physically represented in model.*

**FIGURE 7:**
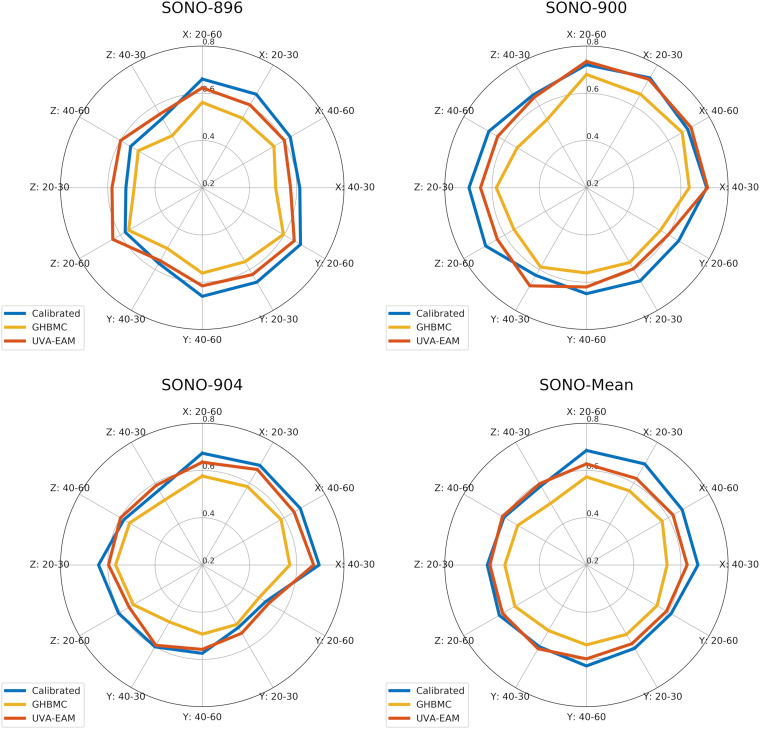
Comparison of wcCORA scores for the calibrated heterogeneous model developed in this study, the GHBMC brain model (Mao et al., 2013), and the anisotropic UVA-EAM model (Wu et al., 2019).

To verify the calibrated material parameters, all 12 impact cases for subjects SONO-896, SONO-900, and SONO-904 were simulated and assessed using wcCORA. The three subject-specific models demonstrated good biofidelity with a mean wcCORA of 0.63 ± 0.06 for all 36 simulations (range: 0.50–0.74). Across the three subjects, SONO-900 demonstrated the highest wcCORA scores (0.68 ± 0.03) and wcCORA was similar for SONO-896 (0.61 ± 0.07) and SONO-904 (0.61 ± 0.06). In general, wcCORA scores were highest in the coronal impacts (0.68 ± 0.04) and similar in the sagittal (0.61 ± 0.06) and axial (0.61 ± 0.06) impacts. A summary of the wcCORA scores for all three subjects is shown in Figure 7. Exemplary nodal displacement-time histories for subject SONO-904 in the 40 rad/s, 60 ms cases are shown in the Supplementary Materials.

The tMRI simulations were run with the final calibrated material model to ensure that the addition of material non-linearity did not influence the strain prediction. As expected, the brain strain response in these simulations was dominated by the infinitesimal shear stiffness, which was optimized in the first stage of material calibration. The incorporation of material non-linearity had negligible effect on the MPS distribution since this second optimization was performed while maintaining the infinitesimal shear modulus previously calibrated.

**FIGURE 8:**
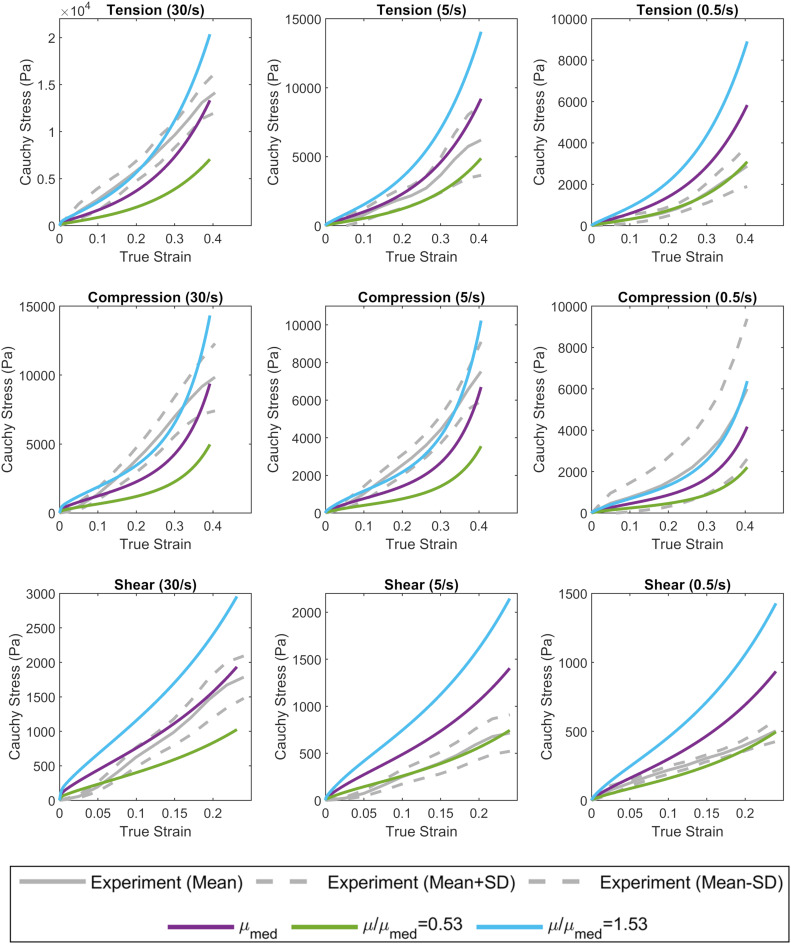
Calibrated material response in tension, compression, and simple shear at rates of 0.5, 5, and 30 1/s compared to experimental material characterization data (Jin et al., 2013). The median (purple), softest (green), and stiffest (blue) response of the brain are shown.

The complex modulus, as a function of frequency, for the median stiffness response and softest (μ/μ_*med*_ = 0.53) and stiffest (μ/μ_*med*_ = 1.53) relative stiffness parts are shown in Figure 4. All three responses were within the range of experimental data. Finally, the median, softest (μ/μ_*med*_ = 0.53), and stiffest (μ/μ_*med*_ = 1.53) material responses were assessed in tension, compression, and simple shear loading at strain rates of 0.5, 5, and 30 1/s and compared to experimental data (Figure 8; Jin et al., 2013). At the highest strain rate (30 1/s) and intermediate (5 1/s), the three material responses were like the experimental corridors for all three loading modes, simultaneously. At the lowest strain rate (0.5 1/s), the three material responses were similar to the experimental in compression but were stiffer in tension and shear.

## Discussion

In this study the heterogeneous CAB-20MSym template model was developed, calibrated, and extensively evaluated using a comprehensive set of experimental data that included measurements of material properties using *in vivo* MRE experiments (Hiscox et al., 2020), *in situ* brain displacement measured using sonomicrometry (Alshareef et al., 2020a), *in vivo* brain strain measured using tagged MRI (Knutsen et al., 2020), and *in vitro* material response (Jin et al., 2013; Meaney et al., 2014). In all assessments of model biofidelity, the CAB-20MSym template model demonstrated a high fidelity to the experimental data which represented a spectrum of TBI severity, ranging from non-injurious (tMRI cases) to moderate-to-severe TBI (sonomicrometry cases).

Our approach to implement heterogeneity using MRE data was selected for several reasons. This technique implemented material heterogeneity without significantly increasing the computational cost. Other studies have implemented heterogeneity using embedded beam elements to explicitly model the structural contributions of axonal fiber tracts (Garimella and Kraft, 2017; Garimella et al., 2019; Wu et al., 2019). However, this technique has been reported to increase the computational cost by a factor of 2.4. For the models developed to calibrate the material parameters, this would have increased the computational time for each simulation from approximately 15 to 36 h (simulating 200 ms of response). As such, the computational time required to run the battery of the 36 *in situ* brain deformation simulations (Alshareef et al., 2020a) would have increased from approximately 540 to 1300 h.

The relative stiffness approach was also chosen with consideration of effects specific to the MRE data. The MRE stiffness measurements we used were determined at a single frequency of 50 Hz, though brain tissue properties from MRE have been shown to be dependent on the actuation frequency due to the viscoelasticity of the brain (Sack et al., 2013). Thus, the specific shear stiffness values recovered at this frequency may not be most relevant for a TBI model. Furthermore, MRE measures tissue stiffness under micron-level displacements. Since the brain is a highly non-linear material, these stiffness results may not be applicable at the finite levels of deformation associated with TBI. Using relative stiffness minimizes any contributions these factors may have had on the absolute stiffness measurements.

There are also several limitations associated with the MRE-based approach. Firstly, although brain heterogeneity was represented as a function of tissue stiffness and not tissue type, the implementation was still isotropic as brain MRE measurements typically assume material isotropy, though methods for extracting anisotropic properties are being developed (Romano et al., 2012; Tweten et al., 2015; McGarry et al., 2020; Smith et al., 2020). Secondly, it was assumed that the relative stiffnesses obtained from the micron-level displacements used in MRE were linear with strain, and that the heterogeneity observed in MRE was applicable for higher levels of deformation. While the validity of these assumptions is unclear, the verification of the brain response with these material parameters over a wide range of rotational loading conditions minimizes their significance.

An inverse FE approach was used to calibrate the material parameters of the heterogeneous CAB-20MSym template model using subject-specific models. However, three challenges faced by any iFE calibration problem are overfitting, obtaining a unique solution, and computational cost. The likelihood of overfitting and obtaining non-unique solutions in an optimization problem is typically increased by using an excessively complicated model (i.e., optimizing many material parameters) and an imbalance between the amount of data used to fit the model and the data used to validate the model (i.e., excessive training data). Furthermore, since one simulation is required for each test case, the computational cost of calibrating a material model increases significantly with the number of parameters included. Given that each simulation in this study took approximately 15 h to run, a robust and efficient approach to material calibration was required.

In the heterogeneous CAB-20MSym template model, each brain parenchyma material requires two parameters to describe bulk properties (density, ρ and Poisson’s ratio, ν), two parameters to describe deviatoric elastic properties (μ and α), and eight parameters to describe viscoelastic properties (*g*_*1*_–*g*_*4*_ and β_*1*_–β_*4*_), for a total of 12 parameters per material. Without further reduction, a full material calibration would require optimizing 120 parameters, which would likely result in non-unique solutions and the computational cost would be prohibitive. In this study, several assumptions were made to reduce the number of material parameters included in the optimization problem. First, all brain tissues were assumed to have the same density and Poisson’s ratio, which is consistent with the TBI biomechanics literature and is common practice in FE brain modeling (Takhounts et al., 2008; Mao et al., 2013; Wu et al., 2019; Alshareef et al., 2020a). This reduced the number of material parameters from 120 to 100. By assuming homogeneous damping and non-linearity, the total number of variables calibrated in this study was reduced to 11 (μ for each material, and a single non-linearity coefficient,α). For the stiffness variables, each material was defined relative to the median value using experimental MRE data, further reducing the total number of calibrated parameters to 2 (μ_*med*_ and α). While non-linearity and viscoelastic properties of the brain have been shown to vary spatially throughout the brain (Johnson et al., 2016; Budday et al., 2017a, b, 2019; Hiscox et al., 2018), these are more challenging measurements and the extent to which viscoelasticity and elastic non-linearity vary remains an open question. These disparities are likely attributed to differences in experimental protocols, including tissue harvest sites, tissue hydration, and loading conditions (Budday et al., 2019). The membranes implemented in the model (falx and tentorium) and CSF parts were not included in the optimization, as a preliminary sensitivity study indicated that the influences of the stiffness and damping of these structures on the deformation response was negligible, compared to the brain parenchyma parts.

We used a combined optimization to calibrate the median shear stiffness and non-linear coefficient, as opposed to an individual calibration for each of the three subjects. The objective of the study was to obtain a singular set of optimal material parameters to be used in the CAB-20MSym template model. Subject-specific material properties could have been combined (e.g., averaged), however, due to the highly non-linear nature of this optimization problem this may not have yielded an optimal solution for the CAB-20MSym template model. Nonetheless, the calibration process indicated that the three subjects likely had different underlying material properties (Figure 5). For example, the estimated optimal median shear moduli for subjects SONO-896, SONO-900, and SONO-904 were approximately 0.70, 1.18, and 2.05 kPa, respectively. A simple average of these values would have resulted in an “optimal” shear modulus of 1.31 kPa, compared to 1.13 kPa determined by the optimization which maximized wcCORA. An important limitation was that only three elderly (57–67 years), cadaveric subjects were used in the optimization process due to the availability of experimental brain deformation data. However, the calibrated model demonstrated a biofidelic response when verified using the *in vivo* tMRI dataset, which included younger, living subjects between 21 and 42 years of age (Knutsen et al., 2020). Nonetheless, the calibrated material parameters may not be representative of the general population and this calibration should be repeated once brain deformation data for more specimens is available. It should also be noted that since these material parameters were obtained using an inverse FE process, they are model specific and cannot be arbitrarily applied to other models (Giudice et al., 2019b). However, given that the calibrated materials have been verified using experimental material characterization data, it is very likely that they represent the underlying material response, and not the contributions of the numerical implementation. Nonetheless, if implemented in another model, the deformation response should be thoroughly investigated prior to model deployment.

The final calibrated material model was verified by its response to experimental tissue data from multiple sources. The complex modulus of the calibrated materials and the response in tension, compression, and simple shear were compared to experimental material characterization data for cortical tissue. In both assessments, the median, softest, and stiffest material responses in the calibrated CAB-20MSym template model were similar the experimental data, verifying that the material parameters obtained from the iFE calibration were physically meaningful and representative of the underlying tissue. In these comparisons, the full range of material response was compared to the experimental data to account for the fact that the experimental data may not necessarily represent the median response of the brain.

The biofidelity of the calibrated CAB-20MSym template model was assessed using brain deformation data from the experiments conducted by Alshareef et al. (2018, 2020a) and Knutsen et al. (2020). Collectively, these datasets encapsulated various magnitudes (2.5–40 rad/s), durations (30–60 ms), and directions of rotational loading (coronal, sagittal, axial). The model demonstrated biofidelic responses under the test conditions used by Alshareef et al. Interestingly, differences in wcCORA scores across subjects reflected the subject-specific relationships between material properties and wcCORA scores in the optimization process. For example, wcCORA scores for SONO-900 were generally greater than those for SONO-896. In the material calibration process, the estimated optimal material properties for SONO-900 were similar to the joint optimal value (μ_0,*med*_ = 1.18 kPa), whereas the estimated optimal material properties for SONO-896 were softer (μ_0,*med*_ ≈ 0.7 kPa). The opposite was true for SONO-904 (μ_0,*med*_ ≈ 2.0 kPa). These differences in calibrated parameters likely reflect variability in material properties between these specimens. While variation in material parameters is to be expected, future work is required to quantify this variability across larger populations. This may be an important factor as subject-specific models of the brain become more prominent in research and clinical applications. Moving forward, the MRE-based framework used to implement material heterogeneity in this study will be adapted to incorporate subject-specific material properties in future models that consider the entire subject-specific brain structure. While the CAB-20MSym template model demonstrated a biofidelic response under the loading conditions investigated in this study, additional verification is required if the model is exercised under loading conditions that deviate from those used in the current study (e.g., blast loading or micron-level harmonic displacements).

While a high-resolution measurement of brain strain at injurious loading conditions is not available, assessing the biofidelity of a brain model’s strain prediction using low severity, non-injurious loading conditions can improve the confidence of a model’s strain response. In general, the CAB-20MSym demonstrated good biofidelity under the axial and sagittal tMRI cases compared to the experimental data. However, there were some discrepancies between the model and experimental results, particularly in the volume fraction exceeding 2% strain. These differences could have been attributed to several factors. Firstly, in the experimental tMRI data set, only resultant head cradle kinematics were recorded. Therefore, to simulate these experiments it was assumed that the head was perfectly coupled to the head cradle and that the applied rotational head kinematics were perfectly uniaxial (either axial or sagittal). While the head was tightly coupled to the head cradle, it is possible that there were slight discrepancies between the head kinematics in the experiments and simulations. Secondly, the brain-skull interface was implemented by modeling the CSF layer between the brain and skull. While many approaches have been investigated to model this interface (Wang et al., 2018), the relative motion between the brain and skull is not well characterized and there is no consensus on best modeling practices for this interface. Therefore, discrepancies between the relative skull-brain motion in the simulations and experiments could have contributed to the observed differences in volume fraction and deformation fields, especially in these low deformation impacts. Furthermore, the brainstem of the CAB-20MSym template model was truncated at the foramen magnum due to a lack of MRE data in the inferior portions of the brainstem and superior portions of the spinal cord. This may have attributed to some of the observed differences, especially in the inferior regions of the brain. However, since the deformations induced in these experiments were small, it is possible that these factors had an exaggerated effect on the predicted strains, and it is not clear how these effects translate to larger deformation cases. Finally, while tMRI is a well-established imaging technique, it can be susceptible to experimental error (approximately 0.7% strain, introduced during filtering or interpolation), which could affect these low strain measurements and the resulting 2% strain volume fractions (Gomez et al., 2019). Nonetheless, the overall biofidelity of the CAB-20MSym template model’s strain prediction under these low severity loading conditions was satisfactory.

### Summary

In this study, the CAB-20MSym template model was developed, calibrated, and extensively verified over a wide range of rotational head kinematic loading conditions. This model utilized a computationally efficient approach for incorporating material heterogeneity that leveraged data from a MRE template image that represented the average brain stiffness of 134 healthy adult subjects. Overall, the developed model demonstrated a biofidelic response for both nodal displacement and element strain metrics. Moving forward this template model will serve as the foundation of the registration-based morphing pipeline developed by Giudice et al. (2020) and can serve as an anatomically accurate model for further investigations of TBI mechanisms and to aid the development of novel protective equipment and safety countermeasures. Furthermore, the framework for implementing material heterogeneity using MRE data can be adapted to incorporate subject-specific material properties in future models of the brain.

## Data Availability Statement

Publicly available datasets were analyzed in this study. This data can be found here: NKI-RS: http://fcon_1000.projects.nitrc.org/indi/enhanced/; MRE-134 Template: https://github.com/mechneurolab/mre134; *In Situ* Brain Deformation: https://www.nhtsa.gov/research-data/databases-and-software.

## Author Contributions

All authors contributed to the conception, design, and interpretation of results presented in this study and are accountable for all aspects of this work.

## Conflict of Interest

The authors declare that the research was conducted in the absence of any commercial or financial relationships that could be construed as a potential conflict of interest.
