# Supplementary material for: Calibration of a Heterogeneous Brain Model Using a Subject-Specific Inverse Finite Element Approach
Source: Front Bioeng Biotechnol. 2021 May 4;9:664268. doi: 10.3389/fbioe.2021.664268 (PMC8129184; doi:10.3389/fbioe.2021.664268)
Supplement: Supplementary file 1 [file Data_Sheet_1.DOCX]

Supplementary Materials

Table SM-1: Numerical implementation of the CAB-20MSym template model.

| Part | Elements (10^3^) | Element Type | Element Formulation | Hourglass Control |
| --- | --- | --- | --- | --- |
| Peripheral CSF | 158 | Voxel  (1 mm^3^) | Constant Stress Element | IHQ = 7^a^ |
| Internal CSF | 102 |  |  |  |
| Brain Parenchyma | 1,165 |  |  |  |
| Ventricles | 14 |  |  | IHQ = 3^b^ |
| Sagittal Sinus | 4 | Quadrilateral  (1 mm^2^) | Belytshko-Tsay Elements | IHQ = 3^b^ |
| Falx | 8 |  |  |  |
| Tentorium | 10 |  |  |  |
| Skull | 101 |  |  | N/A |
| Mid-Sagittal Surface | 17 |  |  | N/A |

^a^IHQ = 7; Linear total strain form of Belytschko-Bindeman assumed strain co-rotational stiffness form

^b^IHQ = 3; Flanagan-Belytshko viscous form with exact volume integration for solid elements

Table SM-2: Constitutive models and material parameters utilized in the preliminary CAB-20MSym template model. All units are in mm, ms, and kg.

| Material | Constitutive Model | Material Parameters |
| --- | --- | --- |
| Peripheral CSF | Linear Viscoelastic | $\rho=1.04\times{10}^{-6} kg/mm^{3}$ |
|  |  | $K=2.19 GPa$ |
|  |  | $G_{0}=5.0\times{10}^{-7} GPa$ |
|  |  | $G_{\infty}=1.0\times{10}^{-7} GPa$ |
|  |  | $\tau=0.0125 ms^{-1}$ |
| Internal CSF and Ventricles | Elastic Fluid | $\rho=1.0\times{10}^{-6} kg/mm^{3}$ |
|  |  | $K=2.1 GPa$ |
| Sagittal Sinus, Falx, Tentorium | Elastic | $\rho=1.13\times{10}^{-6} kg/mm^{3}$ |
|  |  | $E=0.0315 GPa$ |
|  |  | $\nu=0.45$ |

^a^Ogden parameters ($\mu$ and $\alpha$) represent the quasi-static response

Details Regarding *In-Situ* Displacement Experiments and Simulation Setup

In the *in-situ* dataset, sonomicrometry was used to measure 3D *in-situ* displacements of the human brain under dynamic rotational loading of the head. A total of 32 small (2 – 3 mm) and neutrally dense sonomicrometry crystals were implanted in the heads of cadaveric specimens. Of the crystals, 24 were embedded throughout the brain parenchyma (acoustic receivers; “Rx”) and 8 were affixed to the inner surface of the skull (acoustic transmitters; “Tx”). Over the entire loading event, dynamic point-to-point distances were measured between each Rx-Tx pair and the 3D displacement of each receiver was determined using a combination of trilateration and Kalman filtering (Alshareef et al., 2020b). The entire test matrix for each subject included a total of 12 impacts. These included four severity levels, with peak angular velocities of 20 or 40 rad/s over a duration of 30 or 60 ms, applied about the three principal axes of rotation (sagittal, coronal, and axial).

Subject-specific models were generated for subjects SONO-896, SONO-900, and SONO-904. These subjects had MRI imaging performed prior to the specimen preparation and instrumentation. Subject-specific models were generated for each specimen using registration-based morphing to accurately capture the size, shape, and internal anatomy of these subjects (Giudice et al., 2020). The experiments from Alshareef et al. (2020) were simulated by prescribing the actual 6 degree-of-freedom (DOF) experimental boundary conditions to the rigid skull of the subject-specific models. The brain deformation response was assessed by comparing the displacements of each receiver to the displacements of the corresponding nodes in the brain model. All comparisons were subject-specific (i.e., receiver displacements from SONO-896 were compared to nodal displacements from the SONO-896 subject-specific model).

To obtain the most accurate error measurement between the subject-specific models and the experimental data, the coordinates of the initial positions of each receiving crystal were transformed from computed tomography (CT) image space to MRI space using image registration. This ensured that the coordinates of the nodes selected for outputting nodal displacements corresponded to the physical coordinates of the receivers used to measure brain displacement within mesh resolution (approximately 1 mm).

For the SONO-896, SONO-900, and SONO-904 subject-specific models, the morphed volumes were between 0.5 – 8% of the ICV measurements (from segmented CT images) provided in Alshareef et al. (Table SM-3), and the mesh quality was preserved with average element scaled Jacobians between 0.95 – 0.96, characteristic lengths between 0.84 – 0.89, and aspect ratios between 1.23 – 1.30. Minimum scaled Jacobians were between 0.77 – 0.84.

Table SM-3: Intracranial volume (ICV) and mesh quality metrics for subjects SONO-896, SONO-900, and SONO-904. These models are used in the preliminary benchmark assessment and calibration of material properties.

| Subject | ICV* (cm^3^) | Scaled Jacobian (Minimum) | Characteristic Length | Aspect Ratio |
| --- | --- | --- | --- | --- |
| SONO-896 | 1293 (0.5%) | 0.95 ± 0.03 (0.81) | 0.84 ± 0.09 | 1.30 ± 0.19 |
| SONO-900 | 1376 (2%) | 0.95 ± 0.03  (0.77) | 0.85 ± 0.09 | 1.30 ± 0.19 |
| SONO-904 | 1417 (8%) | 0.96 ±0.02  (0.84) | 0.89 ± 0.08 | 1.23 ± 0.14 |

*ICV error computed as percent difference between morphed model and measurement reported in Alshareef et al., which was measured from segmented CT images.

The average error (distance between receiver in MRI and in mesh) between the receiver positions in the MRI image and subject-specific model varied between 0.45 – 0.64 mm, which was within the spatial resolution of the mesh and less than the radius of each receiver (1 mm). Receivers that were within 5mm of the skull were removed from the analysis. Position error and receivers that were excluded from the calibration analysis are summarized in Table SM-4.

Table SM-4: Position error (distance between receiver in MRI space and subject-specific mesh) and excluded receivers for subjects SONO-896, SONO-900, and SONO-904. Receiver locations are depicted with red dots.

| Subject | Position Error (mm) | Excluded Receivers | | | | |
| --- | --- | --- | --- | --- | --- | --- |
| SONO-896 | 0.45 ± 0.19 | Rx-13  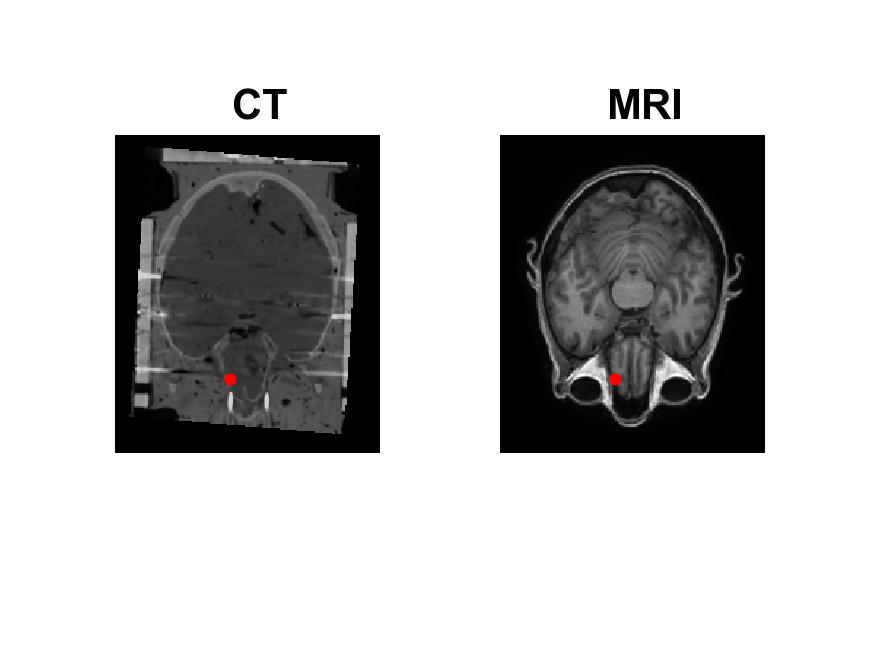 | Rx-26  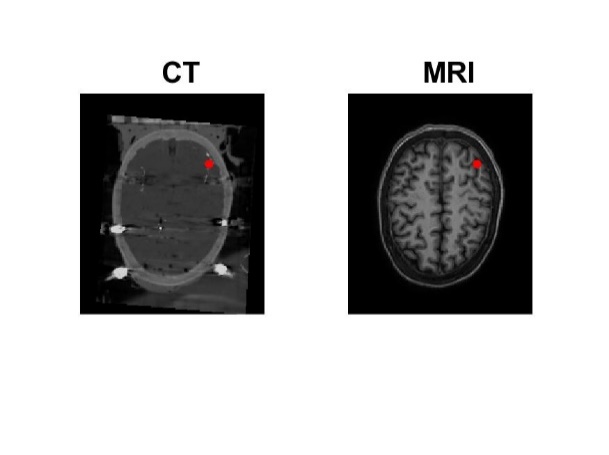 | Rx-30  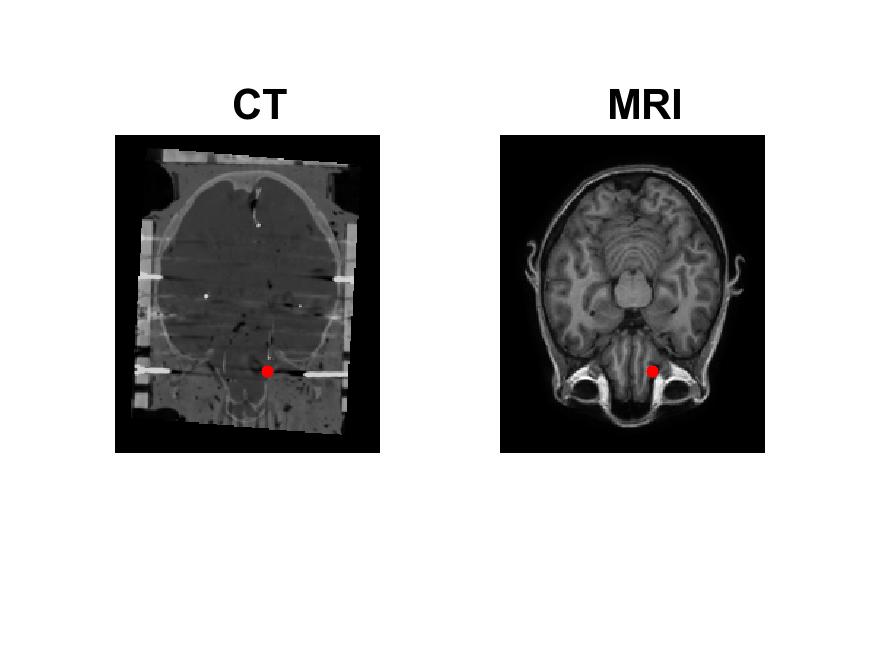 | | Rx-31  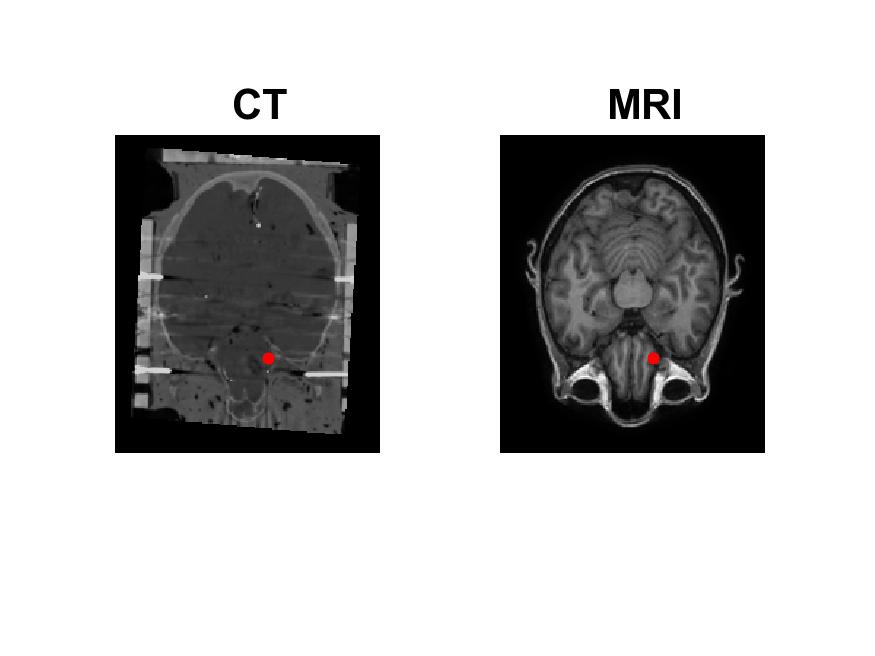 |
| SONO-900 | 0.53 ± 0.20 | Rx-14  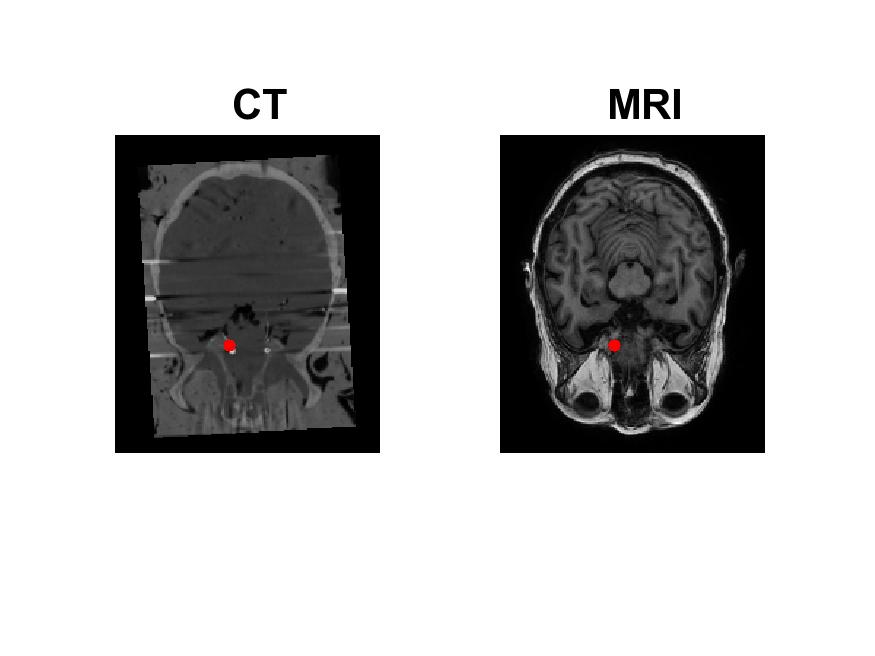 | | | Rx-31  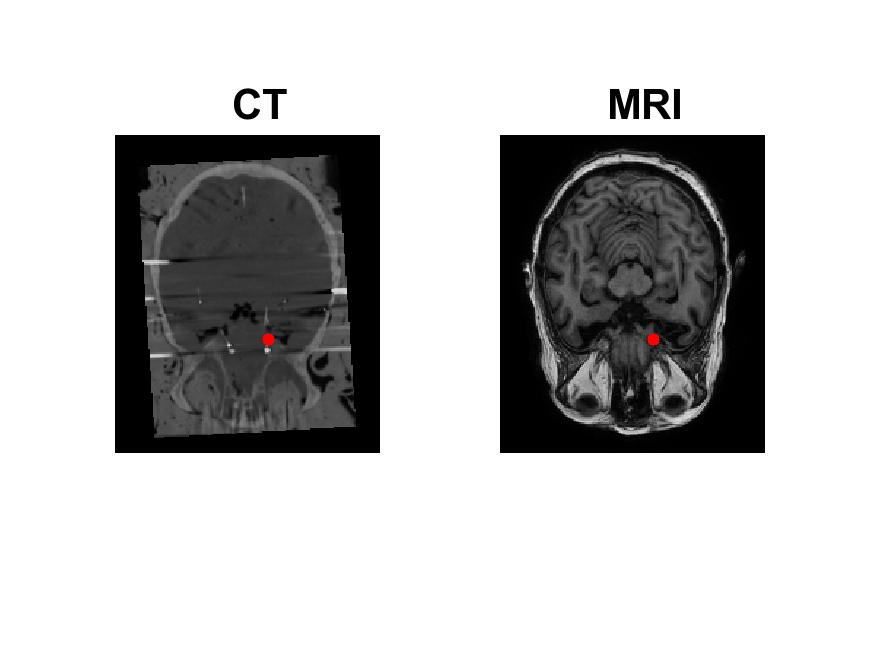 | |
| SONO-904 | 0.64 ± 0.38 | Rx-13  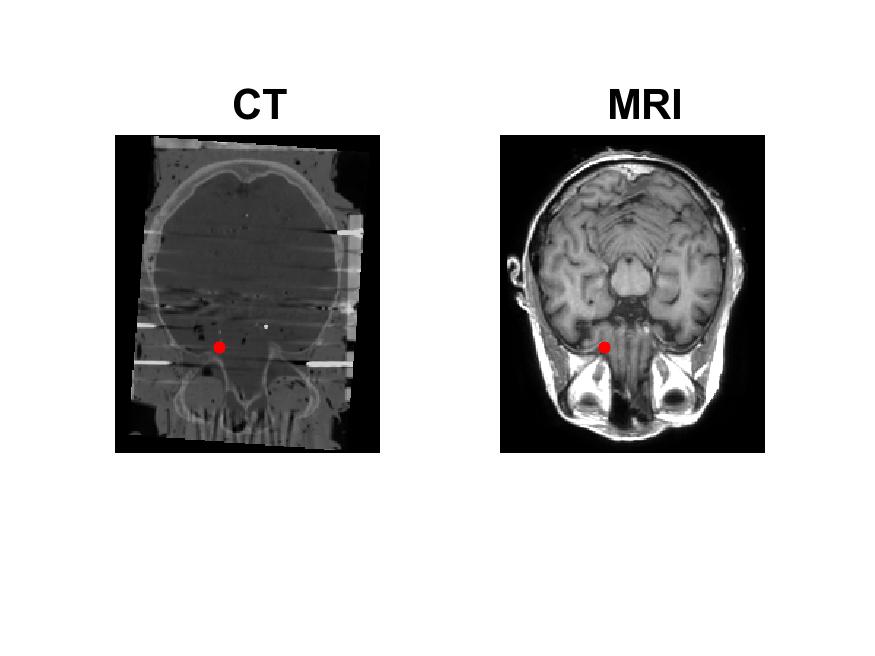 | | | Rx-24  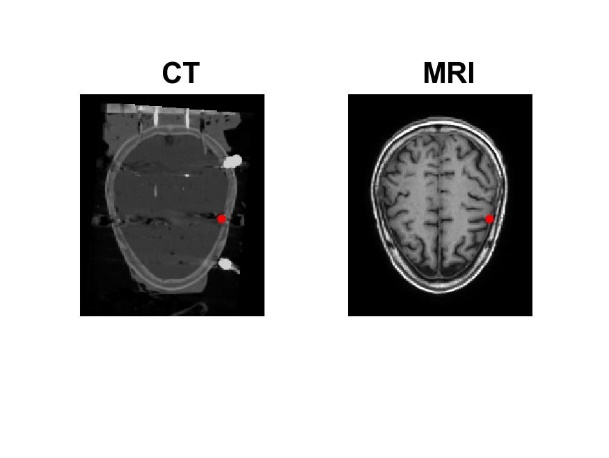 | |

Details Regarding *In-Vivo* Strain Experiments and Simulation Setup

In the *in-vivo* data set, strain response of 5 human volunteers was quantified using tagged MRI (tMRI) during non-injurious rotations of the head (Knutsen et al. 2020). tMRI provides noninvasive measurements of *in-vivo* brain deformation during head rotation by tracking the motion of “tag lines” superimposed on the brain using specialized MRI pulse sequences (Knutsen et al., 2014). Although tMRI studies using human participants are required to be non-injurious and cannot be extrapolated to injurious levels of brain deformation, they provide accurate and repeatable measures of the *in-vivo­* strain response of the brain with high spatial resolution (Knutsen et al., 2014). In this dataset, three subjects (tM-3978, tM-4838, and tM-6176) were subjected to sagittal rotations ($\omega_{max}$ = 1.4 – 1.6 rad/s) and three subjects (tM-3978, tM-7126, and tM-9475) were subjected to axial rotations ($\omega_{max}$ = 4 – 5.4 rad/s) of the head. For the sagittal rotations, the center of rotation (COR) was located at the base of the neck, approximately 21 cm from the superior apex of the skull, and for the axial rotations the COR was located along the midsagittal plane, approximately 7 cm from the posterior apex of the skull. This dataset provided high spatial resolution displacement and strain data, T1-weighted MRI images, and segmentation images.

To assess the strain response, subject-specific models were run using models generated from T1-weighted MRI images of the 5 subjects using registration-based morphing (Giudice et al., 2020). The corresponding head kinematics were prescribed to the rigid dura part about the center of rotation for each subject. All comparisons were subject-specific (e.g., strain data from subject tM-3978 were compared to predicted strains from the tM-3978 subject-specific model).

For the tMRI subject-specific models, the morphed models were within 0.1 – 5% of the ICV measurements (from segmented MRI images) provided in the experimental dataset. Mesh quality was preserved with average element scaled Jacobians between 0.96 – 0.97, characteristic lengths between 0.88 – 0.93, and aspect ratios between 1.18 – 1.22. These volume and mesh statistics are summarized in Table SM-5.

Table SM-5: Intracranial volume (ICV) and mesh quality metrics for the tagged MRI subject-specific models. These models are used in the preliminary benchmark assessment and verification of material properties.

| Subject | ICV* (cm^3^) | Scaled Jacobian  (Minimum) | Characteristic Length | Aspect Ratio |
| --- | --- | --- | --- | --- |
| tM-3978 | 1443 (4%) | 0.97 ± 0.02  (0.85) | 0.91 ± 0.07 | 1.20 ± 0.12 |
| tM-4838 | 1368 (0.1%) | 0.96 ± 0.02  (0.87) | 0.88 ± 0.07 | 1.22 ± 0.13 |
| tM-6176 | 1488 (1%) | 0.97 ± 0.01  (0.90) | 0.92 ± 0.06 | 1.18 ± 0.10 |
| tM-7126 | 1446 (1%) | 0.97 ± 0.02  (0.86) | 0.92 ± 0.06 | 1.19 ± 0.11 |
| tM-9475 | 1536 (5%) | 0.96 ± 0.02  (0.87) | 0.93 ± 0.07 | 1.19 ± 0.11 |

*ICV error computed as percent difference between morphed model and measurement reported in Knutsen et al. (2020), which was measured from segmented MRI images.


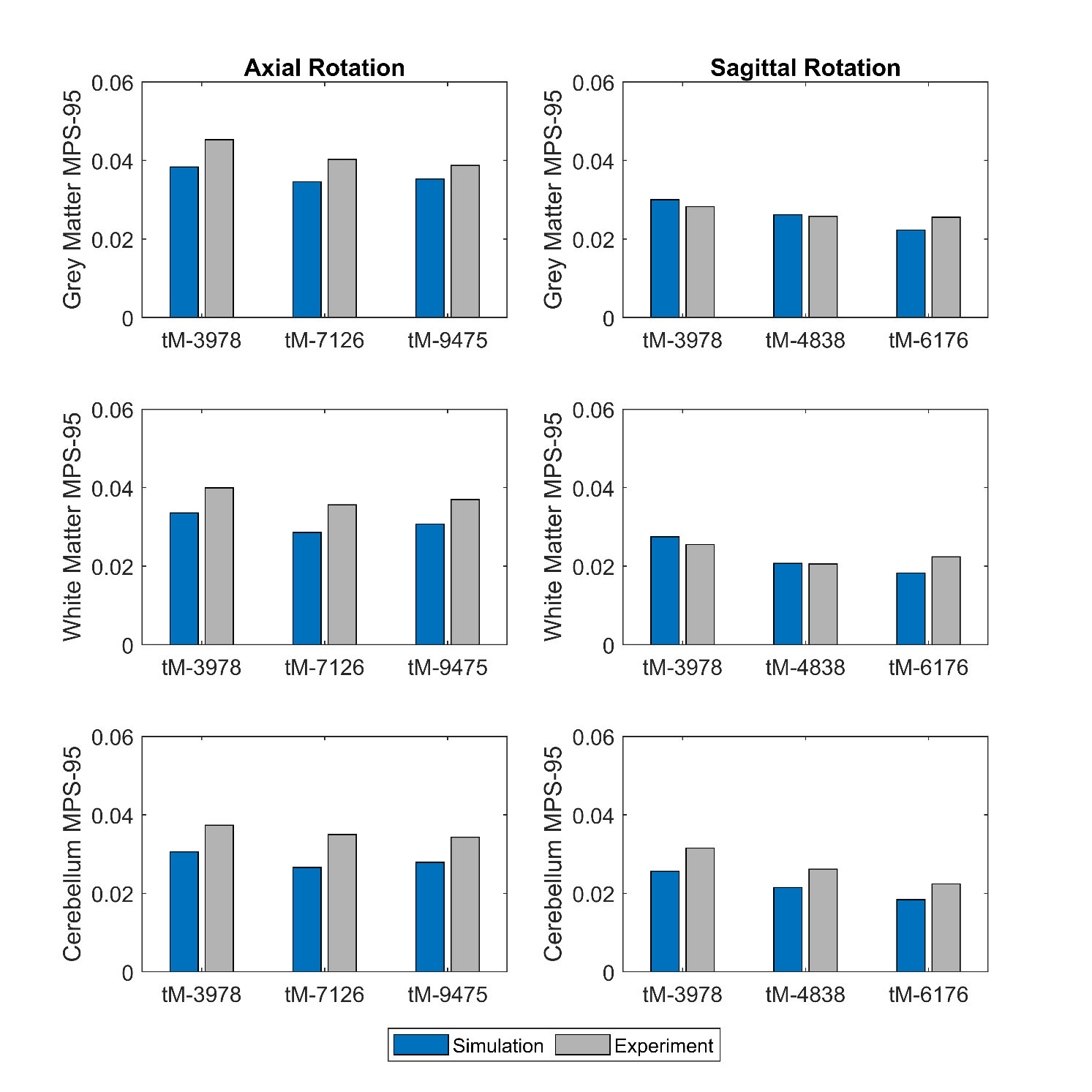


Figure SM-1: Global and regional MPS-95 comparison between calibrated subject-specific models and experimental data (Knutsen et al., 2020).


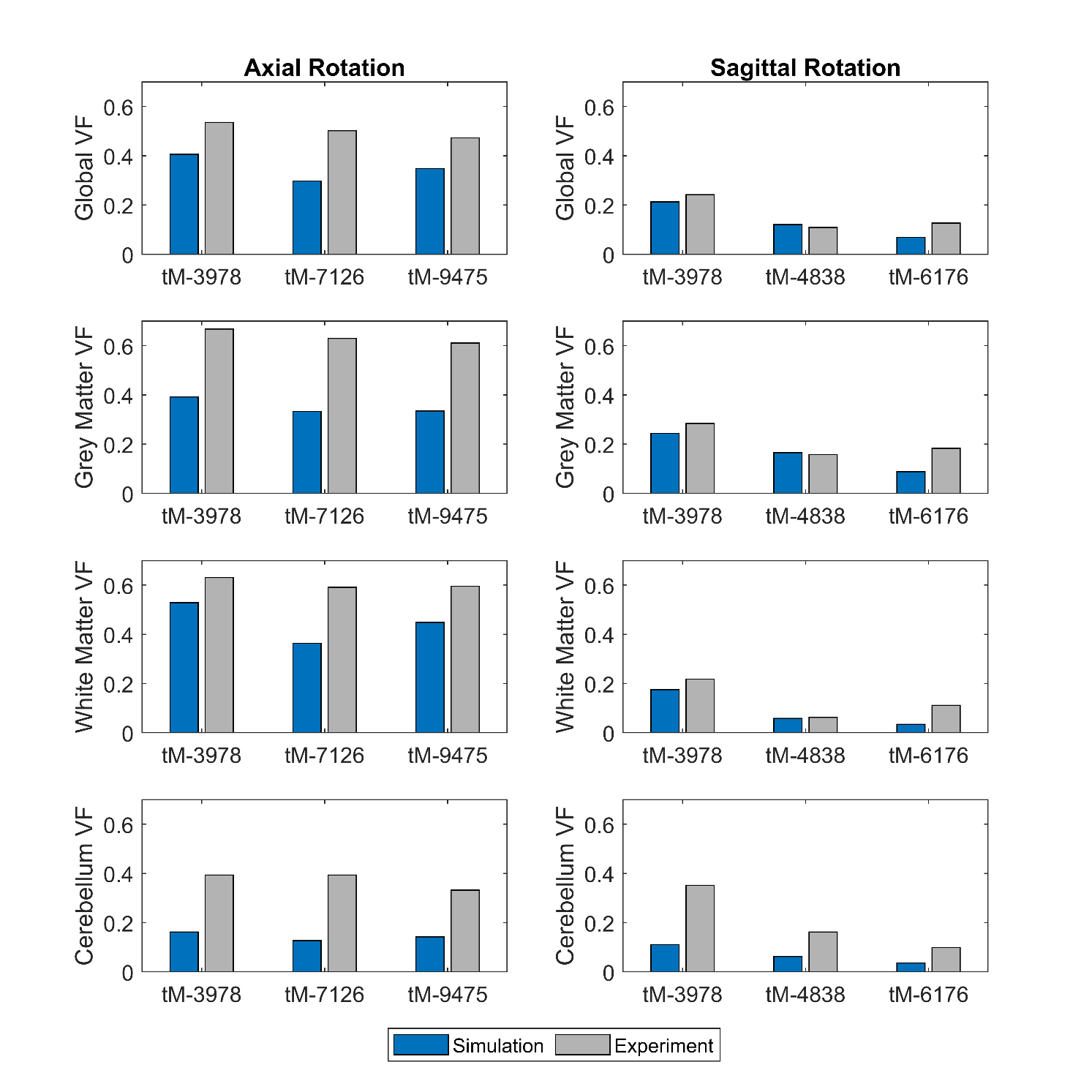


Figure SM-2: Global and regional volume fraction (VF) comparison between calibrated subject-specific models and experimental data (Knutsen et al., 2020).

Supplementary Results from Calibration and Verification of the Nonlinear Coefficient


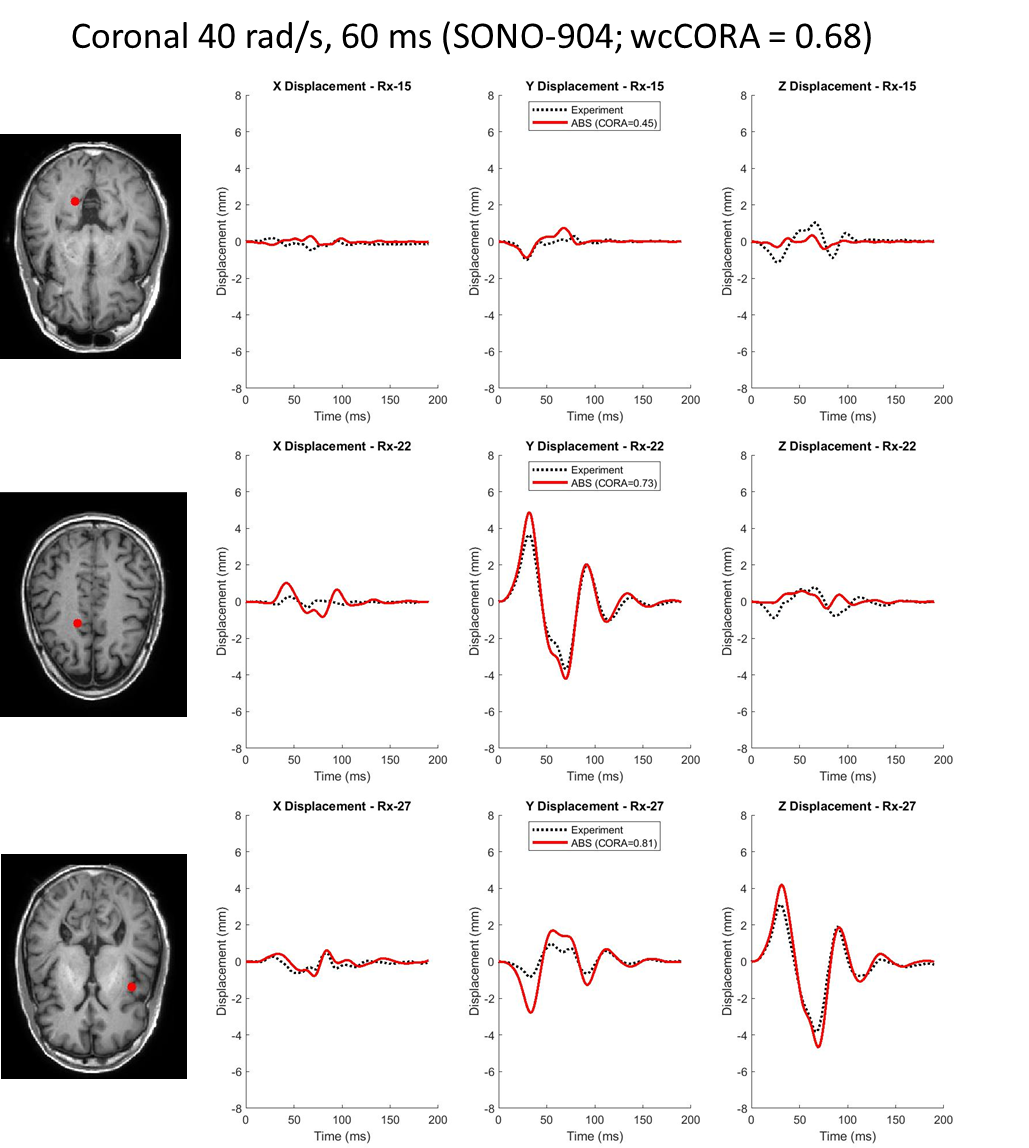


Figure SM-3: Displacement-time histories for receivers 15, 22, and 27 for subject SONO-904 in the Coronal 40 rad/s, 60 ms case. Calibrated model response is shown in red, experimental data is shown in black. The location of the receiver is indicated in the first column.


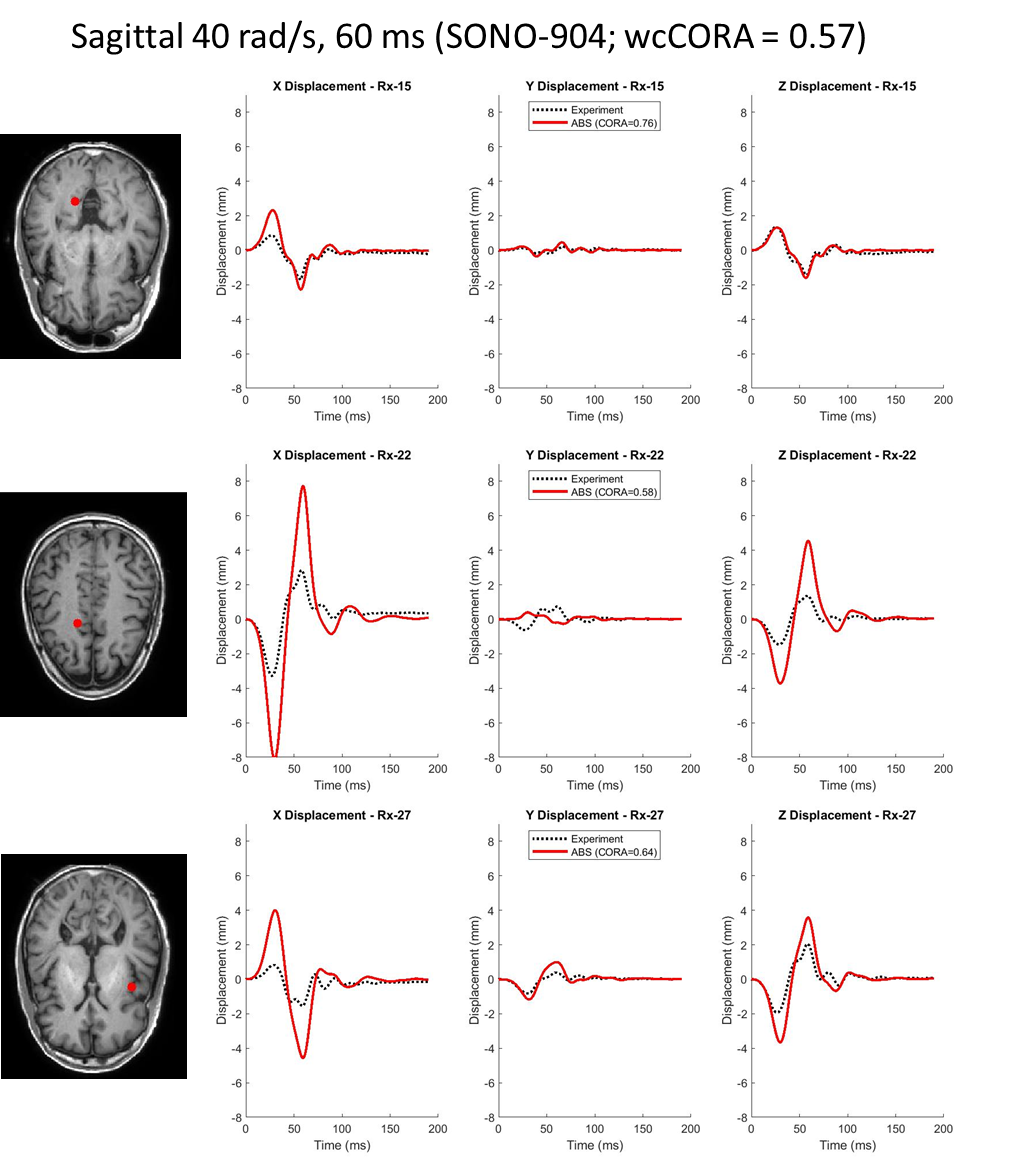


Figure SM-4: Displacement-time histories for receivers 15, 22, and 27 for subject SONO-904 in the Sagittal 40 rad/s, 60 ms case. Calibrated model response is shown in red, experimental data is shown in black. The location of the receiver is indicated in the first column.


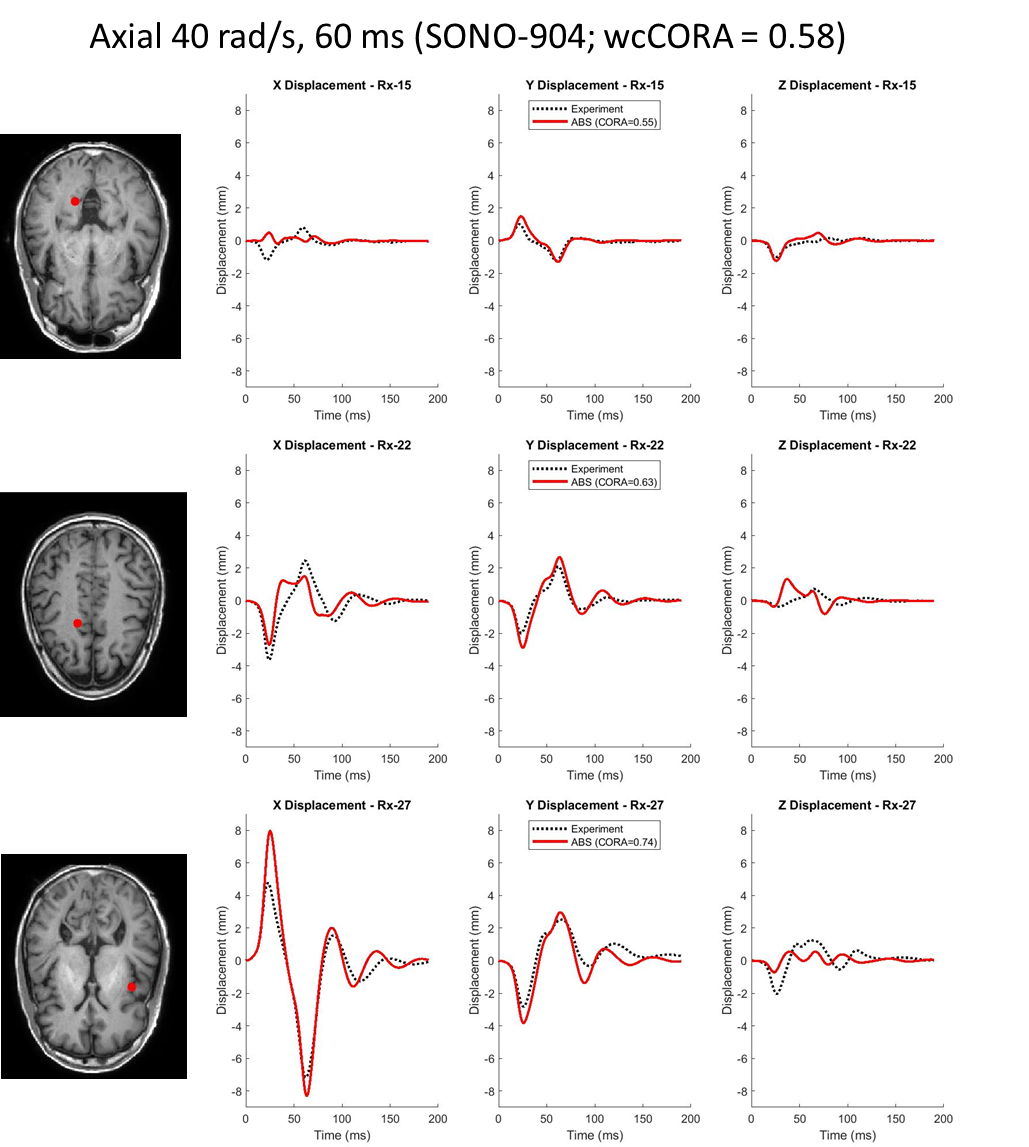


Figure SM-5: Displacement-time histories for receivers 15, 22, and 27 for subject SONO-904 in the Axial 40 rad/s, 60 ms case. Calibrated model response is shown in red, experimental data is shown in black. The location of the receiver is indicated in the first column.
